# Public health round-up

**DOI:** 10.2471/BLT.15.010115

**Published:** 2015-01-01

**WHO report on drowning**

Drowning is a leading and neglected cause of death, according to the *Global report on drowning: preventing a leading killer*. Drowning deaths can be prevented, for example, by fencing off water sources and teaching children – such as this boy living on the edge of an open water source in Manila, the Philippines – to swim. http://www.who.int/violence_injury_prevention/global_report_drowning

**Figure:**
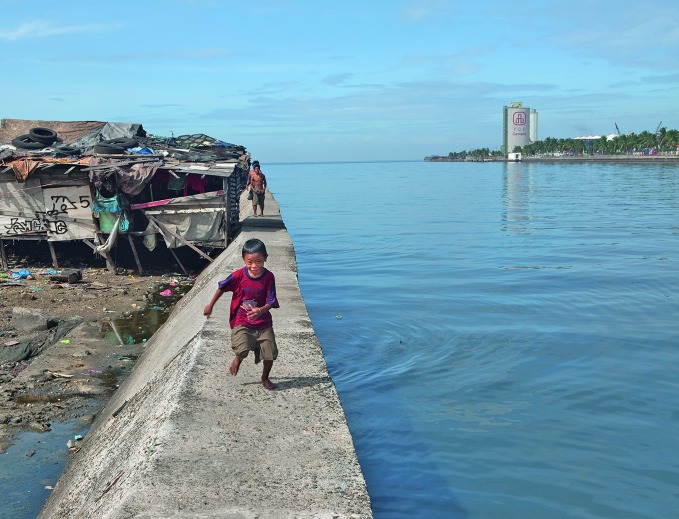
WHO/A Kari

## Preventing cervical cancer 

A guide for health-care providers in health centres and district hospitals to use in preventing and controling cervical cancer includes new recommendations on human papillomavirus (HPV) vaccination and testing. 

The *Comprehensive cervical cancer control: a guide to essential practice*, first published in 2006, has been revised to include scientific evidence published up to December 2013 and was released last month.

The guide includes a new chapter on vaccination against HPV, which can cause cervical cancer. It proposes that girls aged 9 to 13 years should receive two doses of the vaccine, rather than three doses as previously recommended – a change that will save costs for health systems.

The guide also includes a new recommendation to incorporate HPV testing in cervical cancer prevention programmes, a change that has the potential to increase the coverage of screening and improve the quality of national cervical cancer programmes. 

It advises that HPV testing should be offered to women aged over 30 years. However, if HIV prevalence is high, testing should be offered to younger women. If the test result is negative, the woman should not be tested again for at least five years. A positive test result – indicating that she may have a lesion or be at risk of developing cancer in the future – would need further investigation.

HPV testing is being incorporated into cervical cancer prevention programmes in high-resource settings as a primary screening test, but samples need transportation and processing at a laboratory before results can be returned. 

A new low-cost test for HPV infection that can be processed with minimal laboratory infrastructure is being tried out in low-resource settings and may soon be available.

The updated guide highlights the importance of communicating differently around cervical cancer prevention, now that HPV vaccines have been introduced. Instead of focusing on screening for women aged 30 and over, the guide recommends communicating with a wider audience, including adolescents, parents, educators and health professionals, to reach women throughout their lives.

In 2012, an estimated 528 000 new cases of cervical cancer were diagnosed, and 266 000 women died of the disease, nearly 90% of them in low- to middle-income countries. 

Without urgent attention, deaths due to cervical cancer are projected to rise by almost 25% over the next 10 years. 

http://www.who.int/reproductivehealth/publications/cancers/cervical-cancer-guide

## Preventing violence

A new WHO report reviews efforts in 133 countries to prevent violence, including child maltreatment, youth violence, intimate partner and sexual violence, and elder abuse.

According to the *Global status report on violence prevention 2014,* only 47 (35%) of the 133 countries surveyed are implementing large-scale initiatives to prevent violence, such as bullying-prevention programmes, visits by nurses to families at risk, and support to those who care for older people. 

It also found that while 106 (80%) of the 133 countries have enacted a set of 12 laws generally acknowledged to prevent violence, only 76 (57% ) are fully enforcing them.

The report, published by WHO, the United Nations Development Programme, and the United Nations Office on Drugs and Crime, was released last month. 

http://who.int/violence_injury_prevention/violence/status_report/2014

## China’s smoke-free bill

Beijing became the latest and most significant Chinese municipality to ban smoking in public places from June this year.

The move last November in China’s largest city – with some 20 million residents – is seen as paving the way for proposed national legislation to ban smoking in public places all over the country.

The Legislative Affairs Office of China’s State Council published a draft national tobacco control law in the same month which, if adopted, will make all indoor and some outdoor public places in China smoke-free as well as ban tobacco advertising, promotion and sponsorship and require graphic health warnings to cover half of all tobacco packets sold in China.

“We are thrilled to see the Beijing 100% smoke-free law pass, with no loopholes and no exemptions,” said Dr Bernhard Schwartländer, WHO Representative in China. “Beijing has now set the standard for the adoption of a strong set of tobacco control policies at national level.”

“If the draft national regulation is adopted, this will represent unprecedented progress towards China meeting its obligations under the WHO Framework Convention on Tobacco Control (FCTC), and most importantly – dramatic progress towards reducing the epidemic of tobacco-related illness and preventable death in China,” Schwartländer explained.

Beijing is one of 13 cities in China that have passed local smoke-free laws since 2008. China is the world’s largest producer and consumer of tobacco products, with more than 300 million smokers.

http://www.wpro.who.int/china/mediacentre/releases/2014/2014112802

**Cover photo**

A traditional medicine shop in Siamo, Yunnan Province, China.

**Figure:**
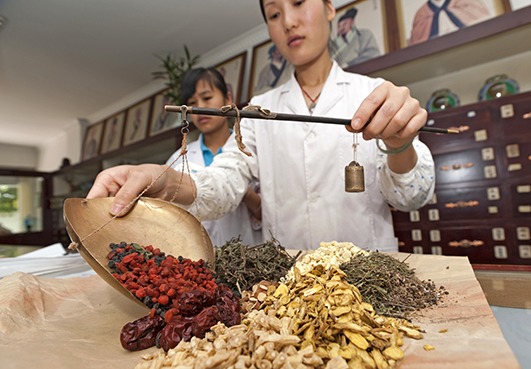
WHO/TDR/Simon Lim

## Gains against malaria

The number of people who died from malaria fell to an estimated 584 000 in 2013 from 882 000 in 2000, according to the *World malaria report 2014*.

Meanwhile, malaria mortality rates – which take into account population growth over time – are estimated to have declined by 47% globally between 2000 and 2013 from 49 to 26 deaths per 100 000 people at risk.

The dramatic fall in malaria mortality is thanks to increased distribution of insecticide-treated bednets, particularly in sub-Saharan Africa, as well as greater access to reliable rapid diagnostic tests and effective treatment, according to the report.

In 2013, almost half of the people at risk of malaria in sub-Saharan Africa had access to an insecticide-treated net, while the number of rapid diagnostic tests procured globally increased to 319 million, up from 46 million in 2008, and a total of 392 million courses of artemisinin-based combination therapies (ACTs) were procured, up from 11 million in 2005.

“The malaria target under Millennium Development Goal 6 has been met, and 55 countries are on track to reduce their malaria burden by 75%, in line with the World Health Assembly’s target for 2015,” said WHO Director-General Dr Margaret Chan in the report’s foreword.

The report notes, however, that funding to combat malaria – at US$ 2.7 billion in 2013 – is only around half of the US$ 5.1 billion needed to fully achieve global targets.

The outbreak of Ebola virus disease in western Africa is hampering malaria control in Guinea, Liberia and Sierra Leone, which are severely affected by the epidemic. At the end of 2013, many inpatient health facilities in those countries remained closed while fewer people sought medical attendance at outpatient facilities for fear of becoming infected with the Ebola virus.

Given the intense malaria transmission in these three countries, WHO has issued new recommendations on temporary measures to control malaria during the Ebola outbreak: to provide ACTs to all fever patients, whether they have been tested or not, to carry out mass anti-malarial drug administration with ACTs in areas that are heavily affected by the Ebola virus and where malaria transmission is high and to distribute insecticide-treated bednets to all areas affected by the epidemic.

http://www.who.int/malaria/publications/world_malaria_report_2014

## HIV recommendations

New WHO recommendations can be used by countries to close important gaps in HIV prevention and treatment services.

The recommendations provide guidance on the prevention of HIV infection in people who have been exposed to HIV infection, such as health workers, sex workers and survivors of rape, and on preventing and managing common opportunistic infections, such as tuberculosis and pneumonia, and other severe bacterial infections in people infected with HIV.

The guidelines are published as a supplement to WHO’s 2013 consolidated guidelines on the use of antiretrovirals.

http://www.who.int/hiv/pub/guidelines/arv2013/december2014supplementARV.pdf

**Looking ahead**

**26 January–3 February – WHO Executive Board meeting in Geneva, Switzerland. **http://www.who.int/mediacentre/events/2015/eb136

**26–31 January – Prince Mahidol Award Conference, Bangkok, Thailand**. http://www.pmaconference.mahidol.ac.th/

**4 February – World Cancer Day. **http://www.who.int/nmh/events/2014/world-cancer-day

**12–14 February – The First World Congress on Ear and Hearing Care, New Delhi, India. **http://www.sh2030worldcong.org

**14–18 March – 3rd World Conference on Disaster Risk Reduction, Sendai, Japan. **http://www.wcdrr.org

**20 March – World Oral Health Day. **http://www.worldoralhealthday.com/fdi-launches-its-world-oral-health-day-2015-smile-for-life-campaign

**22 March – World Water Day** This year marks the end of the International Decade for Action: Water for Life 2005–2015. http://www.who.int/water_sanitation_health/decade2005_2015

